# Clinical intervention of a tighter-than-tolerated fit of aesthetic hand and finger prosthesis via controlled silicone swelling: A novel, speedier and versatile alternative method

**DOI:** 10.1097/MD.0000000000030885

**Published:** 2022-10-07

**Authors:** Michael E.L. Leow, Lan Anh T. Le, Yiong Huak Chan, Alphonsus K.S. Chong

**Affiliations:** a Department of Hand and Reconstructive Microsurgery, National University Hospital, Singapore; b Department of Hand and Reconstructive Microsurgery, National University Hospital, Singapore; c Biostatistics Unit, Yong Loo Lin School of Medicine, National University of Singapore, Singapore; d Department of Hand and Reconstructive Microsurgery, National University Hospital, Singapore; e Department of Orthopaedic Surgery, Yong Loo Lin School of Medicine, National University of Singapore, Singapore.

**Keywords:** aesthetic finger prosthesis, controlled silicone swelling, elastomer expansion, prosthetic fit rectification, residuum circumference, silicone hand prosthesis

## Abstract

A tighter-than-tolerated fit of aesthetic hand prosthesis is conventionally rectified by stretching the affected segment to plastic deformation. This method is not only time-consuming, but also ineffective in stretching irregular, non-cylindrical prosthesis segments apart from the “wrist and digits”.

This study investigates controlled silicone swelling as an alternative method of expanding aesthetic hand and finger prosthesis to address a tight fit.

The technique of “controlled” swelling that minimizes oil uptake to as little as is necessary to achieve the desired magnitude of elastomer expansion was demonstrated using experimental test samples. Brush-coats of a cosmetics-grade oil, KF-96A-10CS, 2 a time, were applied on Cosmesil^TM^ samples to obtain elastomer expansion. The same technique of staggered oil delivery was used on tight-fitting segments of patients’ prosthesis, with test-fitting of each incremental expansion till satisfactory outcomes were achieved. Percentage circumference increases in swelled test samples and in all rectified/ patients’ prostheses were then compared to validate the effectiveness of the method.

Circumference increases in the test samples after each 2 coats were significantly different (*P* <.001). Representative (unreinforced) Samples 1, 2, and 3 recorded circumferential increases of 4.0% to 11.4% within 30 minute after swelling by 2.6% to 9.7% from 2 to 6 oil coats. This largely correlated with patient data, where circumferential increases of 3.6% to 9.5% from 2 to 6 oil coats were collectively recorded in all fit-rectified finger prostheses. Swelling in the expanded proximal segment of all 24 finger prostheses was estimated to be within 9.7%. Of these, 22 (92%) required 2 to 4 oil coats, inferring a lower still swelling of 6.5%. The rapid and consistent elastomer expansion enabled prosthetic fit rectification to be achieved in a much shortened time. Clinical outcomes indicated that low swelling magnitudes involving small amounts of 2 to 6 brush-coats of oil have no adverse effects on the prostheses. None of the participating patients had reported any incidence of discernible change in all of the important properties of their prostheses.

Outcomes based on the Cosmesil^TM^-KF-96A-10CS elastomer-oil combination demonstrated that controlled silicone swelling involving minimal use of oil is an effective method of intervention for a tighter-than-tolerated prosthetic fit of silicone hand and fingers.

## 1. Introduction

Aesthetic silicone prostheses play a useful role in the holistic rehabilitation of patients with hand amputations.^[1–3]^ They are custom-molded to fit on the residuum via suction-cup effect.^[4]^ The elasticity of silicone rubber allows an airtight cupping of the residuum such that an incipient slippage of the prosthesis immediately creates a vacuum effect that checks further displacement. A good fit, defined as one that is secure and without pressure pain, is achieved primarily by making the circumference of the prosthesis slightly smaller than that of the stump.

It is important that techniques in the fabrication and fitting of silicone prostheses, which are vastly different from that of functional prostheses, are able to resolve prosthetic fit issues that can arise over the course of the patients’ rehabilitation. Clinical intervention to address a common loose fit problem is by adding layers of silicone on the inside of the prosthesis, which effectively reduces its inner circumference, tightening fit. For more recent amputations therefore, a slightly tighter fit is routinely planned into the prosthesis to accommodate residuum shrinkage from receding edema and late tissue atrophy that can take many months, sometimes years to stabilize, particularly when reconstruction, flaps, and scars are involved. For most patients, the initial tighter fit is a non-issue. A small group may, however, require minor fit adjustments for optimal comfort early post fitting. Conversely, a late tight fit can arise due to weight gain as a result of lifestyle changes and long-term medications.

For digital and transcarpal fittings, a tighter-than-tolerated fit is conventionally rectified by expanding the affected proximal segment by stuffing in a cylindrical “prosthesis expander” (a rigid tube larger in size) and leaving it in place long enough to achieve plastic deformation. This method is highly time-consuming, as it can take a day or 2 of uninterrupted stretching to expand a fully vulcanized silicone prosthesis. Another constraint of significance with this method is its limited effectiveness in stretching non-cylindrical prosthesis segments such as the “knuckles” and “thenar eminence”, as opposed to the “digits and wrist”. This presents a challenge in partial/reconstructed hand fittings involving irregular-shaped residual limbs where minor localized expansion is occasionally needed for targeted pressure relief over bony prominences, raised flaps or hypertrophic scars adherent on the knuckles. It should be pointed out that test-fits undertaken as part of the prosthesis fabrication process are never foolproof in averting post-production fit rectifications. Not infrequently, patients may feel no discomfort during test-fits, but can later experience pressure pain after donning their prosthesis longer or using their hands more actively in real-life settings – thus the necessity for effective techniques of intervention for addressing a tight fit.

All silicones will readily adsorb oil and swell irreversibly as a consequence, with the oil becoming part of the elastomer structure. This phenomenon is known as silicone swelling.^[5,6]^ Its dimensional change effect is generally undesirable, but a necessity in various applications. Because elastomers can swell in a controlled way in selected oils and solvents, the phenomenon has been exploited in applications such as swellable seals in the oil recovery, automotive, medical tubing, and electronics industries.^[7–9]^ Silicone swelling presents opportunities for prosthetic applications, which the authors have exploited in this study.

This study investigates controlled silicone swelling as a method of expanding aesthetic hand and finger prosthesis for rectifying a tight fit. The technique of “controlled” swelling that minimizes oil uptake to as little as is necessary to achieve the desired expansion was first demonstrated using experimental samples. Results from test samples were then compared against retrospective patient data to validate the effectiveness of the method.

## 2. Materials and methods

### 2.1. Experimental test samples

**Figure 1.**
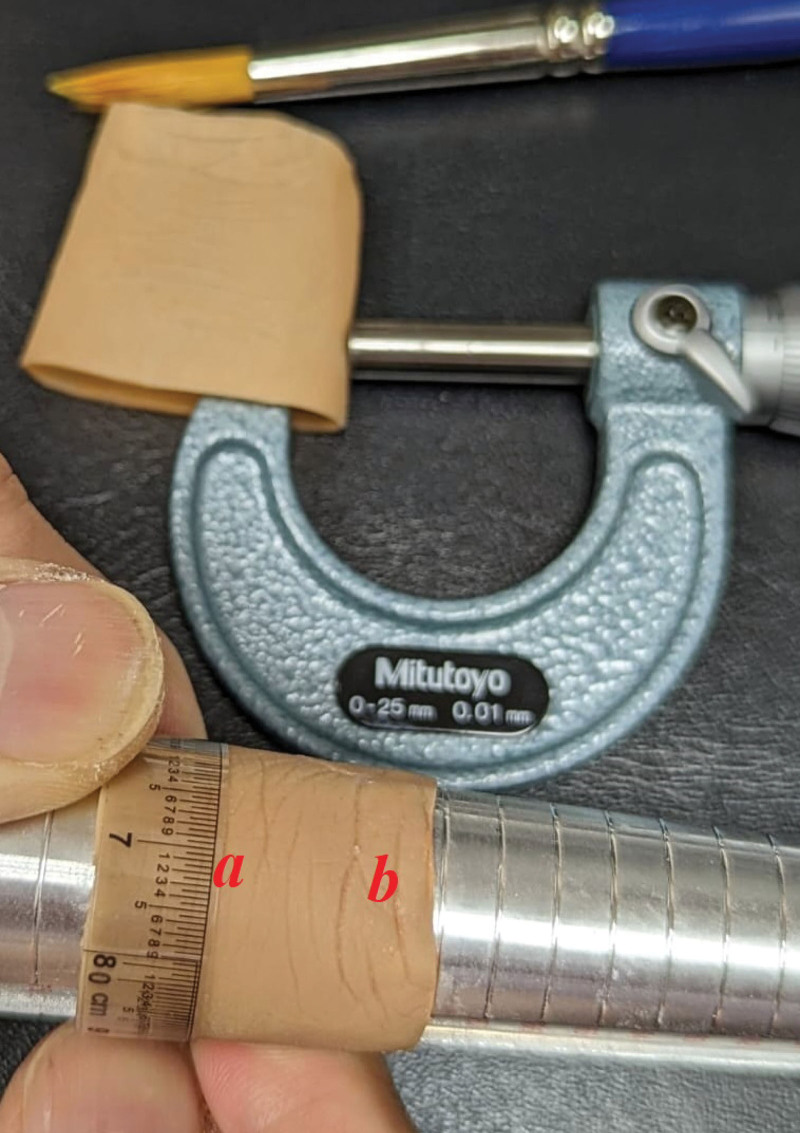
Outer circumference measurements of each sample were taken at points “*a*” and “*b*” using a soft precision measuring tape. Care was taken to ensure both ends of the tape – the “0” mark and the reading – were in alignment when taking measurements. In addition, a goldsmith ring sizer was slotted through the test sample to maintain circular form for each measurement.

Methods for this experimental part of the study were based on digital fitting for through-PIPJ (proximal interphalangeal joint) amputations (Fig. 1). Four test samples, designated as Sample 1, 2, 3, and 4 (Table 1), were made from the same finger mold using a layered molding methodology previously reported.^[10,11]^ Each sample was molded in double layers of different shades using a medical-grade silicone (Cosmesil^TM^, Principality Medical Ltd., UK). Samples 1, 2, and 3 were planned identical in order to test for consistency of results. Sample 4 had an intermediate layer of nylon stockinet sandwiched in between for tear strength enhancement. A sample thickness of 0.8 mm was planned so as to be representative of the average thickness of patients’/fitted prostheses, which varies from 0.75 mm to 0.85 mm (according to individual preferences). Consistency in thickness was achieved by maintaining a constant solvent/silicone ratio in the molding mixture. All samples were cured for 48 hours to full strength. Final mean sample thickness (of 5 measurements taken at different points using a micrometer screw gauge), was recorded (Table 1).

**Table 1:** Percentage circumference increase and percentage swelling of expanded test samples vs. number of oil coats/immersion in oil bath.

Samples		Controlled swelling				Immersion in oil bath till constant equilibrium weight
		Circumference & weight				
	*Thickness*	*Baselin*e	*2 coats*	*4 coats*	*6 coats*	
	Mean ± SD	(before)	(10 min)	(20 min)	(30 min)	(3 h)
S1-*a*	0.82 ± 0.01	75.0	78.5	81.0	83.0	99.0
S1-Δ*a*			3.5	6.0	8.0	24.0
S1-P*a*			4.7 %	8.0 %	##10.7%	32.0%
S1*-b*		70.0	73.0	75.5	77.5	92.0
S1-Δ*b*			3.0	5.5	7.5	22.0
S1-P*b*			#4.3%	7.9%	10.7%	31.4%
S1-*w*		1.54	1.59	1.64	1.69	2.92
S1-P*w*			3.2%	6.5%	**9.7%	89.6%
S2-*a*	0.81 ± 0.02	75.0	78.0	81.0	82.5	98.0
S2-Δ*a*			3.0	6.0	7.5	23.0
S2-P*a*			#4.0%	8.0%	10.0%	30.7%
S2*-b*		70.0	74.0	76.0	78.0	93.5
S2-Δ*b*			4.0	6.0	8.0	23.5
S2-P*b*			5.7%	8.6%	##11.4%	33.6%
S2-*w*		1.56	1.60	1.65	1.70	2.98
S2-P*w*			*2.6%	5.8%	8.9%	91.0%
S3-*a*	0.78 ± 0.01	75.0	78.5	81.5	83.0	98.5
S3-Δ*a*			3.5	6.5	8.0	23.5
S3-P*a*			#4.6%	8.6%	10.7%	31.3%
S3*-b*		70.0	74.5	76.5	78.0	94.5
S3-Δ*b*			4.5	6.5	8.0	24.5
S3-P*b*			6.4 %	9.3%	##11.4%	35.0%
S3-*w*		1.52	1.57	1.61	1.66	2.89
S3-P*w*			3.3%	5.9%	9.2%	90.1%
S4-*a*	0.8 ± 0.01	75.0	77.5	80.0	82.5	98.0
S4-Δ*a*			2.5	5.0	7.5	23.0
S4-P*a*			#3.3%	6.7 %	##10.0%	30.7%
S4*-b*		70.0	72.5	74.5	76.5	91.0
S4-Δ*b*			2.5	4.5	6.5	21.0
S4-P*b*			3.6%	6.4%	9.3%	30.0%
S4-*w*		1.65	1.70	1.75	1.80	2.90
S4-P*w*			3.0%	6.1%	9.1%	75.8%

SD = standard deviation.

*a, b* = circumference across points *a* and *b* of Sample 1, 2, 3, and 4, rounded down to nearest 0.5 mm.

∆*a*, ∆*b* = absolute circumferential increase across point *a* and *b* (mm).

P*a*, P*b* = percentage circumferential increase across point *a* and *b*.

*w* = weight of test samples in grams (g).

P*w* =* percentage swelling = percentage weight increase*.

#,

## = Lowest, highest % circumferential increase after 2 to 6 oil coats in each sample.

* ,

** = Lowest, highest % swelling after 2 to 6 oil coats (collectively amongst Sample 1, 2, and 3).

Only the proximal segment that is relevant to prosthetic fit was studied. All samples were cut through the PIPJ to exclude the uninvolved distal segment. Outer circumferences were measured at points “*a*” and “*b*” (identified by characteristic creases) using a soft precision measuring tape (30 cm/0.5 mm, Eyhiu Shop, People’s Republic of China). For accuracy, care was taken to ensure both ends of the transparent tape – the “0” mark and the reading – were in alignment when taking measurements. A goldsmith ring sizer was used to maintain circular form for each circumferential measurement.

A silicone oil – KF-96A-10CS (Shin-Etsu Chemical Co. Ltd., Japan) – that is widely used as ingredients in cosmetics, sunscreens, hair lotions and quasi-drugs (see Supplemental Digital Content, http://links.lww.com/MD/H436, which shows the technical data/applications of KF-96A-10CS oil) was chosen as the swelling agent. This oil was selected after pilot trials conducted in the early days of our research into aesthetic silicone prostheses. There has been no reported incidence of skin irritations from its widespread and long-time use in the skincare industry.

A coat of the KF-96A-10CS oil was evenly applied all around each sample using a soft-bristle brush (ColorPro, Size 6, Japan). A fresh coat was brushed on after 5 minute – this was observed to be the time taken for the complete adsorption of each preceding coat when the sample surface is dry to touch and without traces of oil. Baseline circumferences and sample weights, and their increases after each 2-oil-coats/10-minute interval were measured (Table 1). The number of coats were limited to 6 in the context of making minor prosthetic fit rectifications. For circumferences, the median of 3 readings taken were recorded. Readings were rounded down to the nearest 0.5 mm. To minimize intra-operator bias, all readings were verified by an independent person not involved in this study.

#### 2.1.1. Relationship between *percentage swelling, percentage weight increase* and *percentage circumference increase*

Silicone swelling is quantified in terms of the *percentage weight increase* of the swelled specimen as a consequence of oil uptake.^[5–7]^ Percentage swelling (P*w*) of test samples after adsorption of each 2-oil coats was computed as follows:


$$\begin{array}{l} Pw=\frac{Absolute\;weight\;increase}{Initial\;sample\;weight}\times 100\% \end{array}$$

By definition, *percentage swelling* and *percentage weight increase* are the exact same parameter. However, *percentage swelling* and *percentage circumference increase* are 2 different, but related parameters. Swelling results in expansion – more specifically, circumferential increase, which directly influences prosthetic fit. Each elastomer-oil combination exhibits swelling behavior that is unique to itself (this is dependent on their solubility parameters) and can vary from slight to a few 100%. A 100% swelling means a weight increase by 100% of the swelled specimen. A silicone sample that is immersed in an oil bath will continue to uptake oil and expand till a point of equilibrium is reached when it can no longer adsorb oil, and when weight of the swelled specimen remains constant. This may be referred to as “uncontrolled swelling”. For preservation of elastomer properties, it is desirable to have outcomes with as small a percentage swelling and as high a percentage circumference increase.

#### 2.1.2. Equilibrium state, anti-staining, and elasticity tests

Following the 6^th^ oil coat, a simple anti-staining test was performed. A ballpoint pen was used to make markings on each sample to test if they erased completely after 30 minute of staining. After conclusion of the anti-staining test, all 4 samples were submerged in a KF-96A-10CS oil bath and monitored half-hourly till equilibrium. The samples were then removed from the oil bath. A simple, nonstandard elasticity/tension test was finally conducted whereby each sample was manually stretched, donned and doffed over prosthesis expanders of progressively larger sizes.

### 2.2. Retrospective patient data

This study has been approved by our hospital’s institutional review board (National Healthcare Group Domain Specific Review Board 2021/00766) under the waiver of informed consent category, which requires deidentification of all patient data. Six hundred twenty records of patients fitted with aesthetic silicone prostheses from 2002 to 2018 were reviewed retrospectively to identify those cases that had fitting rectifications via the swelling method. These included digit and partial/total hand prostheses expanded to relieve pressure on the proximal phalanx, wrist, flaps, knuckles or radial/ulnar-side flare of the residuum. All prostheses were molded in the same manner as the test samples from the same Cosmesil^TM^ silicone. Tight-fitting segments were expanded using the same KF-96A-10CS oil in likewise 2 coats-manner, with test-fits in between till satisfactory fit loosening was achieved for the patient. For wrist expansion, a larger brush (ColorPro, Size 10, Japan) was used to apply the oil coats due to the larger surface area involved. Pre- and post-rectification outer circumference of all finger prostheses and other relevant data were retrieved.

Standard use and care advice provided to patients at final fitting included temporarily not wearing their prosthesis when there are rashes or bruises on their residuum, and to promptly send their prosthesis for repairs if they noted any tears. Patients were reviewed at 6 months to check for any residuum issues/properties change in their prosthesis using a questionnaire (Table 2). Feedback was gathered via phone calls that guided patients through each of the 10 scenarios covered in the questionnaire, which essentially encompass all the important attributes/properties of aesthetic prostheses.

**Table 2:** Questionnaire monitoring (accelerated) change in the residuum and prosthesis at 6 months.

Scenarios	Any signs/noticeable change?
*Properties/attributes of prosthesis*	
1. Wear/thinning of outer layer	Yes/No
(evidenced by loss of surface creases)	
2. Minor tears around proximal edge	Yes/No
3. Loss of elasticity (less stretchable)	Yes/No
4. Loss of suppleness (increased stiffness)	Yes/No
5. Loss of color-match (discoloration)	Yes/No
6. Gets stained easily (e.g., by inks, clothing dyes)	Yes/No
7. *Oil leach from prosthesis	Yes/No
*Condition/health of residuum*	
8. Incidence of skin rashes/irritations	Yes/No
9. Appearance of a macerated skin	Yes/No
10. Prosthesis reverted to tight fit	Yes/No

* As characterized by an oily prosthesis surface or excessively oily skin around the residuum.

### 2.3. Statistical analysis

Statistical analysis was performed using Statistical Package for Social Sciences version 26.0 (SPSS Inc., Chicago, IL) with statistical significance set at 2-sided 5%. Descriptive statistics for numerical variables were presented as mean ± SD. Paired t test was used to compare continuous variables of the same samples before and after oil coatings.

## 3. Results

### 3.1. Experimental test samples

#### 3.1.1. Percentage circumference increase vs percentage swelling

**Figure 2.**
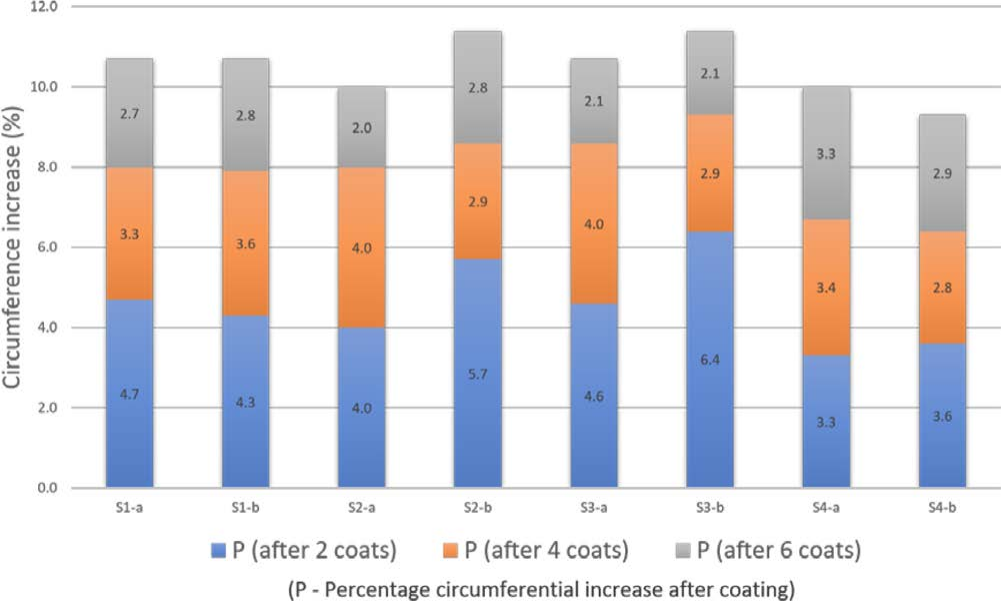
Chart showing percentage circumferential increases in the test samples through points “*a*” and “*b*” after 2, 4 and 6 oil coatings.

Rapid, consistent and largely uniform swelling and circumferential increases were recorded in all experimental samples after each 2 oil-coat (Table 1, Fig. 2). The increases in circumference after each 2 coats were significantly different (*P* < .001, Table 3). Circumferential increases were smaller for nylon-reinforced Sample 4 compared with all 3 non-reinforced samples. There were no significant differences in percentage circumference increase between points “a” and “b” after 2, 4 and 6 coats, with mean differences of between 0.23% to 0.85% (*P* > .05).

**Table 3:** Percentage circumference increases accumulated after every 2 oil coats of all 4 samples at points “*a*” and “*b*”.

Number of coats	N	Mean ± SD	Median (interquartile)	Range	Pair t test
2	8	4.6 ± 1.04	4.45 (3.70–5.45)	3.3–6.4	P (2 coats)–baseline: *P* < .001
4	8	7.9 ± 0.97	8.0 (7.0–8.6)	6.4–9.3	P (4 coats)–P (2 coats): 3.4 ± 0.5, *P* < .001
6	8	10.5 ± 0.72	10.7 (10.0–11.23)	9.3–11.4	P (6 coats)–P (4 coats): 2.6 ± 0.5, *P* < .001

P = percentage; SD = standard deviation.

#### 3.1.2. Equilibrium state, stain resistance and elasticity

In the initial swelling phase from 2 to 6 oil coats, percentage swelling was consistently lower than percentage circumferential increases. However, this reversed at some point as swelling continued toward equilibrium, with the test samples showing increasing resistance to expansion.

All 4 samples reached equilibrium after 3 hours in oil bath, attaining maximum swelling of 89.6% (Sample 1), 91.0% (Sample 2), 90.1% (Sample 3) and 75.8% (Sample 4), but with circumferential increases that ranged from 30.0% to 35%.

**Figure 3.**
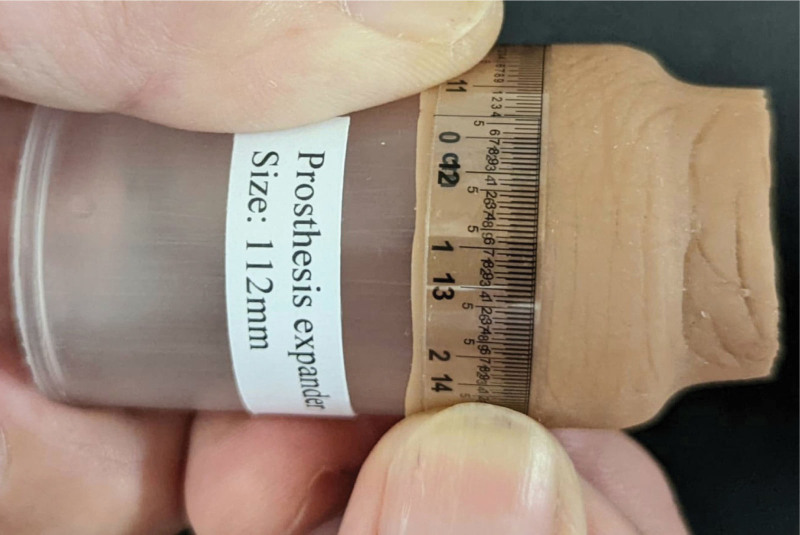
Test Sample 1 being stretched further on a “prosthesis expander” to a circumference of 116.5 mm (subsequent to it attaining maximum equilibrium circumference of 99 mm at point “*a*”).

All ink markings made on the test samples (after the 6^th^ coat) erased completely by mere rubbing off with the bare thumb. As shown in Figure 3 for Sample 1, all samples stretched further with ease from their respective maximum/equilibrium circumference to 116.5 mm and reverted to their pre-stretch sizes without yielding (i.e., plastic deformation) or any discernible loss of elasticity. No attempt was made to stretch the samples beyond this size to the point of rupture.

### 3.2. Retrospective patient data

#### 3.2.1. Circumference and rectification time

Twenty seven cases were identified comprising 18 finger cases involving 24 finger prostheses, and 9 hand cases involving 9 partial/total hand prostheses. Five cases – 3 finger and 2 hand – were returning patients for tight fit issues as a result of weight gain and medications for chronic conditions. The rest were newly fitted cases with post‐fitting time ranging from 2 to 10 weeks. All patients had expressed urgency to have their prosthesis back in the same visit and accepted that for this to be possible, a different method that involved the use of oil to expand rubber had to be used.

Even numbers of 2, 4 or 6 coats were used for expanding finger prostheses, whereas hand prostheses had 4 to 6 coats. The distribution of oil coats used, prosthesis/involved side, frequency of usage and outer circumference data, and rectification time (64 ± 16 minutes, range 45–90) were as shown in Table 4. Prosthesis use was grouped as daily, weekends or occasional. Patients wore their prosthesis mostly for social outings and for light-duty work in sales and administrative vocations. Out of 24 finger prostheses rectified, 9 had 2 oil coats, 13 had 4, with the remaining 2 having 6 coats (Table 5). Except for 2 wrist circumferences, pre- and post-expansion data were unavailable for the remaining hand cases, as it was infeasible to delineate/measure, for example, expansion over the “knuckle” and other non-cylindrical prosthesis segments. All hand prostheses had nylon reinforcement as opposed to finger prostheses and were slightly thicker for structural integrity.

**Table 4:** Distribution of oil coats used for expanding patients’ prostheses versus circumference increase to achieve satisfactory fit loosening.

Patient no.	Prosthesis digit (affected hand)	Prosthesis usage	Circumference before expansion (mm)	No. of oil coats	Circumference after expansion (mm)	% Circumferential increase	Rectification time** (min)
**Finger cases**							
1	Index (L)	Daily	50.0	2	52.0	4.0%	45
2	Index (R), middle (R)	Daily	56.0, 57.5	2, 2	58.0, 60.0	#**3.6%,** 4.4%	60
3	Index (R)	Daily	60.0	4	64.0	6.7%	60
4	Little (R)	Weekends	50.0	2	52.0	4.0%	45
5	Middle (L), ring (L)	Daily	56.0, 55.5	4, 4	60.0, 59.5	7.1%, 7.2%	75
6	Little (R)	Weekends	43.0	2	45.0	4.7%	45
7	Middle (R), ring (R)	Daily	61.0, 58.0	4, 2	65.5, 61.0	7.4%, 5.2%	75
8	Middle (R)	Daily	55.0	4	59.0	7.3%	45
9	Index (R), middle (R)	Daily	63.0, 63.5	4, 4	67.5, 68.5	7.1%, 7.9%	75
10	Middle (R)	Daily	46.0	2	48.0	4.3%	45
11	Thumb (R)	Weekends	60.0	6	65.5	9.2%	50
12	Index (L)	Daily	45.0	2	47.0	4.4%	45
13	Index (L)	Occasional	55.0	4	59.5	8.2%	60
14	Ring (R)	Daily	48.0	2	50.0	4.2%	45
15	Little (L)	Weekends	50.0	4	54.0	8.0%	45
16	Index (R), middle (R)	Weekends	62.0, 63.0	4, 6	67.0, 69.0	8.1%, ##**9.5%**	75
17	Index (R), middle (R)	Daily	54.5, 55.0	4, 4	59.0, 59.0	8.3%, 7.3%	75
18	Middle (L)	Daily	54.0	4	58.0	7.4%	45
**Partial/reconstructed hand cases**							
19	Transmetacarpal (R)	Daily	180.0 (wrist)	6	197.0	9.4%	90
20	Congenital (R)	Daily	132.0 (wrist)	6	144.0	9.1%	90
21	Replantation (L)	Daily	[Segment, knuckle]*	6	-	(Satisfactory)	75
22	Partial hand (R)	Weekends	[Segment, knuckle]	4	-	(Satisfactory)	75
23	Toe transfer (R)	Weekends	[Segment, flap]	4	-	(Satisfactory)	75
24	Toe transfer (L)	Occasional	[Segment, flap]	4	-	(Satisfactory)	75
25	Reconstruction (L)	Daily	[Segment, bulky flap]	6	-	(Satisfactory)	90
26	Partial hand (R)	Daily	[Segment, radial flare]	4	-	(Satisfactory)	75
27	Reconstruction (L)	Occasional	[Segment, radial flare]	6	-	(Satisfactory)	75

(R) = right; (L) = left; [segment, xxxxx]

* – non-cylindrical prosthesis segment expanded where it was not feasible to measure circumference.

** Includes time taken in performing test-fits in between the 2^nd^, 4^th^ and 6^th^ oil coats.

#,

##Lowest, highest percentage circumferential increase (collectively amongst all fit-rectified finger prostheses).

**Table 5:** Range of percentage circumference increase – experimental versus patient data.

	Number of coats	N	Range
Samples 1, 2, and 3	2	6	#4.0–6.4
(unreinforced, including	4	6	7.9–9.3
“a” & “b”)	6	6	10.7–##11.4
Fit-rectified finger	2	9	#3.6–5.2
prostheses	4	13	6.7–7.9
	6	2	9.2–##9.5

#,

##Lowest and highest.

#### 3.2.2. Patients’ feedback on residuum and prosthesis

All patients, except 1 lost to follow-up (Patient 22), monitored their residuum and prosthesis as advised and provided feedback. None reported any incidence of change in their residuum and prostheses (Table 2).

## 4. Discussions

Most patients are able to tolerate a slightly tight prosthetic fit, which is deliberate and planned into the fabrication to accommodate late stump shrinkage. However, in 4% of our cases, the prosthesis needed minor expansion to loosen fit for optimal comfort. The need for speedier outcome and a more versatile method prompted us to look into alternative techniques of intervention. Controlled silicone swelling was investigated in this regard.

Our results with test samples have shown that silicone swelling in oil produces dimensional change that is consistent and proportional. This is in line with its application in the craft of display models/figurines-making where exact replicas in larger sizes are obtained by swelling the original silicone moulds in spirits or oils (https://youtu.be/P6fnGUL307U). It is important to note that unlike swelling in volatile solvents, which is reversible as the solvent evaporates from the elastomer, oi-induced expansion is irreversible. The consistent and permanent swelling effects of silicone elastomers presents opportunities for application in limb prosthetics, but has never been exploited in any previous reports. In this study, controlled silicone swelling using a selected elastomer-oil combination – Cosmesil-KF-96A-10CS – was exploited in a novel, quicker, and versatile method of expanding silicone prosthesis for rectifying a tighter-than-tolerated fit. The method entails staggered application of brush-coats of oil on affected prosthesis segments, followed by test-fitting each incremental expansion till a satisfactory loosening of fit is achieved for the patient. Our early trials have established that the Cosmesil-KF-96A-10CS combination produces the largest circumferential increase with least oil uptake. This selected silicone-oil combination, together with the technique of staggered delivery in brush-coats, limits oil uptake to as little as is necessary to produce adequate circumferential increase. Table 1 showed that the percentage circumferential increases from 2 to 6 oil coats within 10 to 30 minute was 4.3% to 10.7%, 4% to 11.4% and 4.6% to 11.4% for Samples 1, 2, and 3 respectively (all without reinforcement and representative of patients’ prostheses). In clinical practice, the recorded magnitudes of circumferential increase are enough to translate to a satisfactory release. This was corroborated by the collective outcomes for all rectified finger prostheses, for which a largely correlated circumferential increase of 3.6% to 9.5% was recorded (Table 4) – after accounting for differences in prosthesis thickness. With 2 to 6 oil coats, rectification time (including time for test-fits) was no more than 60 minutes per digit prosthesis. Compared with over a day of uninterrupted stretching, the rapid circumferential increase obtainable with the swelling method allows for “immediate, in-between” assessment of each incremental fit adjustment, enabling outcomes to be achieved in a dramatically shortened time. This is of practical significance, as it makes within-the-hour rectification and return of prosthesis a reality, with savings of a return trip for patients in addition to minimizing prosthesis use downtime. Of equal importance is that this method presented an effective solution to tackling pressure points on non-cylindrical stump segments in partial/reconstructed hand fittings. With this method, brushes of oil coats are applied directly on the “knuckle” or any tight-fitting prosthesis segment to effect localized elastomer expansion, thereby easing pressure over specific trouble areas. The method is equally effective for expanding nylon-reinforced prostheses, as demonstrated by a similar ease of elastomer expansion in reinforced Sample 4, as well as satisfactory outcomes achieved for all rectified hand prostheses. Lamination with nylon fabric increases tear resistance, but also makes the prostheses a little stiffer. The smaller circumferential increases in the reinforced sample could be attributed to the scaffolding effect of the nylon layer, the presence of which also possibly explained its lower equilibrium swelling of 75.8% (S4-Pw, Table 1). Given that sample thickness was maintained at 0.8 mm, there is a correspondingly smaller elastomeric mass available for oil uptake.

A logical question when swelling silicone prosthesis is whether it will change or weaken its properties. This is, however, a point of concern only when elastomers are swelled to an “over-expanded” state from excessive oil uptake. Overexpansion is a valid concern regardless of method, whether by stretching or swelling. However, in the context of making minor fitting adjustments in this study, this concern is adequately addressed by controlling/minimizing elastomer swelling to as little as is needed. Our results showed that elastomer expansion needed to translate to satisfactory outcomes involve low magnitudes of swelling, requiring no more than 6 oil coats – a small enough amount to be inconsequential to the prostheses other than the dimensional change. This was corroborated both by experimental results and the absence of patient reports with regard to any deterioration in the elasticity, suppleness, surface details, color-match, anti-staining, as well as wear and tear properties of their prosthesis at 6 months (Table 2). This monitoring timeframe served to exclude/limit the confounding factor of normal wear and tear in the prosthesis, which normally surface at around 9 months on average with daily use.^[12,13]^

In attempting to quantify the percentage swelling that occurred “in the expanded proximal segment” of all rectified finger prostheses, their range of circumferential increase was compared against that of experimental samples (Table 5). Only data from non-reinforced Samples 1, 2, and 3 was used for comparison to exclude the extraneous factor of nylon-reinforcement. Notably, a 2-to-6 oil coats produced circumferential increases that ranged from 4.0% to 11.4% (through point “*a*” and “*b*” taken together) with percentage swelling of 9.7% at the highest (Table 5, S1-P*w*, Table 1). In comparison, the highest percentage circumferential increase recorded amongst all expanded finger prosthesis was 9.5% (Table 4). We therefore estimate that swelling in the expanded segment of all finger prostheses would be within 9.7%. Notably, 22 (92%) of a total 24 finger prostheses required only 2 to 4 oil coats to achieve satisfactory outcome. This would infer a lower still swelling of 6.5%. It bears reiterating again that the smaller the swelling, the better it is for preservation of elastomer properties. Of note, the experimental results also demonstrated the resilience of the swelled Cosmesil^TM^ silicone at much higher swelling magnitudes. At the extreme end, for instance, Samples 1, 2, and 3 reached equilibrium swelling of 89.6% (S1-Pw), 91.0% (S2-Pw) and 90.1% (S3-Pw) respectively after 3 hours in oil bath. It was remarkable that despite having attained maximum circumferences of over 98 mm, Samples 1, 2, and 3 could stretch further on prosthesis expanders to 116.5 mm (i.e., 156% of its original circumference) without tearing or yielding (Fig. 3).

### 4.1. Limitations

Due to the gaps in pre- and post-rectification circumferences – other than for two cases involving the wrist – the satisfactory outcomes achieved for seven partial/total hand cases could not be validated by patient data. Nonetheless, based on our clinical experience, patients are generally sensitive to compression pressure and are usually able to tell if adequate release is achieved in the prosthetic fit. Also, it would be of interest to conduct standard, more extreme tensile studies on swelled samples to help shed light on whether there is any “intangible” change in the elastomer, notwithstanding the low swelling magnitudes involved. This is, however, beyond the scope of the present study. Such investigations will need collaboration with materials engineers and could be undertaken as further work. More importantly, outcomes of this study indicated that any intangible elastomer change, even if present, is inconsequential in the practical context of normal everyday use of silicone prostheses with no discernible effects on all of their important properties.

The current study was initiated after frequent patient requests for shorter wait times and took a balance approach that considered any potential concerns vis-à-vis the efficacy and benefits of the swelling method for effecting prosthetic fit rectifications. The fact that aesthetic prostheses are worn for social occasions and light duties was also a factor considered. Subject to prior preliminary tests, this method could potentially be extended to similar prosthetic application using other proprietary brands of silicone and oil.

## 5. Conclusions

Outcomes based on the Cosmesil-KF-96A-10CS elastomer-oil combination demonstrated that controlled silicone swelling is an effective alternative technique of intervention for addressing a tighter-than-tolerated fit of aesthetic hand and finger prostheses. Minimal application of 2 to 6 brush-coats of oil with swelling magnitudes of 6.5% to 9.7% is adequate for making minor fit adjustments. The technique of staggered brush-on application of oil, followed by test-fits, provides safeguards against larger-than-necessary expansion to preserve elastomer integrity, as opposed to uncontrolled swelling in oil bath.

## Acknowledgments

The authors wish to thank Mr Ambrose Lim, Senior Technician, for assisting in the molding procedures and in the verification of all measurements taken in this study.

## Author contributions

**Conceptualization:** Michael E.L. Leow.

**Data curation:** Michael E.L. Leow.

**Formal analysis:** Michael E.L. Leow, Anh T. Le, Yiong Huak Chan.

**Investigation:** Michael E.L. Leow.

**Methodology:** Michael E.L. Leow.

**Project administration:** Anh T. Le.

**Supervision:** Alphonsus K.S. Chong.

**Validation:** Anh T. Le.

**Visualization:** Michael E.L. Leow.

**Writing – original draft:** Michael E.L. Leow.

**Writing – review & editing:** Michael E.L. Leow, Alphonsus K.S. Chong.

## Supplementary Material


