# Supplementary material for: Clinical intervention of a tighter-than-tolerated fit of aesthetic hand and finger prosthesis via controlled silicone swelling: A novel, speedier and versatile alternative method
Source: Medicine (Baltimore). 2022 Oct 7;101(40):e30885. doi: 10.1097/MD.0000000000030885 (PMC9542914; doi:10.1097/MD.0000000000030885)
Supplement: Supplementary file 1 [file medi-101-e30885-s001.pdf]

Shin-Etsu

Technical Data Sheet

KF-96A-10cs－5,000cs

version 2014/9/19
Shin-Etsu Chemical Co., Ltd.

KF-96A-10cs－5,000cs
Silicone Fluid

Silicone fluid (Middle viscosity)

■ Features & Advantages

- Smooth texture
- Good spread
- Water-repellency
- Skin protect

■ Applications

- Hair care
- Skin care
- Make-up
- Sunscreen

■ Percentage of use 0.1-50 %

■ INCI name DIMETHICONE

■ Structure

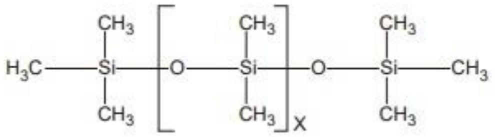

■ Characteristics

Properties	Unit	10cs	20cs	30cs	50cs	100cs	200cs	300cs	350cs	500cs	1,000cs	3,000cs	5,000cs	Standard method / condition
Appearance (color)	—	Colorless												Visual observation
Appearance (transparency)	—	Transparent												Visual observation
Appearance (texture)	—	Liquid												Visual observation
Percentage of active raw material	%	<30	<5	<1.5	<0.5									150°C × 24 h
Viscosity	mm^2/s	10	20	30	50	100	200	300	350	500	1,000	3,000	5,000	Cannon fenske viscosimeter
Specific gravity	—	0.935	0.950	0.955	0.96	0.965	0.970	0.970	0.970	0.970	0.970	0.970	0.975	Densimeter
Refractive index	—	1.399	1.400	1.401	1.402	1.403								Refractometer

(Not specific values)

KF-96A-10cs－5,000cs
